# Support and service provision for women with substance use issues exiting prison in Australia: findings from a 10-year retrospective cohort analysis

**DOI:** 10.1108/IJOPH-02-2025-0015

**Published:** 2025-09-09

**Authors:** Layla Maree Edwards, Sacha Kendall Jamieson, Reem Zeki, Sungwon Chang, Julia Bowman, Craig Cooper, Elizabeth Sullivan

**Affiliations:** Faculty of Health, University of Technology Sydney, Sydney, Australia; Social Work and Policy Studies, Faculty of Arts and Social Sciences, Sydney School of Education and Social Work, The University of Sydney, Sydney, Australia, and School of Public Health, Australian Centre for Public and Population Health Research, University of Technology Sydney, Sydney, Australia; Research Unit, Justice Health and Forensic Mental Health Network, Malabar, Australia, and School of Medicine and Public Health, The University of Newcastle, Callaghan, Australia; Centre for Improving Palliative, Aged and Chronic Care through Clinical Research and Translation (IMPACCT), Faculty of Health, University of Technology Sydney, Sydney, Australia; Research Unit, Justice Health and Forensic Mental Health Network, Malabar, Australia; Drug and Alcohol Services, Justice Health and Forensic Mental Health Network, Malabar, Australia; College of Health, Medicine and Wellbeing, The University of Newcastle, Callaghan, Australia, and Custodial Health, Justice Health and Forensic Mental Health Network, Malabar, Australia

**Keywords:** Women prisoners, Problematic drug use, Post-release care, Gender-responsive, Housing, Employment

## Abstract

**Purpose:**

The purpose of this study is to inform gender-responsive policy and practice by investigating services and supports that women with substance use issues need when exiting prison.

**Design/methodology/approach:**

This is a retrospective cohort study of women (n = 989 / 18%) and men (n = 4609 / 82%) exiting prison who engaged with the prison health program “Connections” in Australia between 2008 and 2018. Using pre-release assessment data, six re-entry variables were computed: substance use, mental health, housing, employment, social support and parenting. These variables represent areas of support/service provision that might be needed on release. Descriptive statistics were calculated. Associations between “re-entry support needs” and sex were estimated by multiple logistic regression, adjusting for potential confounders.

**Findings:**

“Re-entry support need” was higher for women in housing (52% vs 46%), employment (88% vs 79%) and parenting (68% vs 60%) compared to men. Multivariate analysis found women also had increased odds of “re-entry support need” in these areas. Similar proportions of women and men were sentenced for substance-related offences (74.4% vs 75.3%), yet, twice the proportion of women were incarcerated for the first time (20.4% vs 8.6%), and double were sentenced to ≤6 months compared to men (44.3% and 22.9%).

**Originality/value:**

This is a large-scale study of women’s re-entry needs on exiting prison, which is rare. The findings of this study indicate that gender-responsive policy/practice must account for women having higher level of need in housing, employment and parenting and ceasing the use of punitive short-term sentences. Gender-responsiveness requires addressing women’s social determinants of health and incarceration via policy, service provision and practice that will support women and address broader gender inequity taking an intersectional approach.

## Background

Women’s rates of imprisonment have grown significantly in the past decade in Australia, increasing by 33% from 2014 to 2024 (ABS, 2024) and worldwide (a global increase of 60% from 2000 to 2022) (Penal Reform International, 2023). This growth has been partly attributed to high recidivism rates (Yukhnenko *et al.*, 2019; Bell *et al.*, 2020). In Australia, half (48%) of women in prison haven been previously incarcerated (AIHW, 2020). In Australia, more women are sentenced for illicit drug offences than for any other criminalised offence (ABS, 2024). Drug-related arrests in the USA have increased nearly 216% for women, compared to only 58% for men over the past 35 years (Herring, 2020). Women tend to serve shorter sentences (less than 12 months) than men, which is a reflection of the minor, non-violent crimes they have been sentenced for (e.g., drug possession, petty theft) (AIHW, 2020; Gelb, 2010; Philippe, 2020; Querengässer and Traub, 2020). This punitive criminal legal system response is highly disruptive and harmful to women’s lives, especially when considering that most incarcerated women (61%) are mothers, and their imprisonment has a significant impact on their dependent children (Walker *et al.*, 2019; Kennedy *et al.*, 2020). A substantial number of women are on remand (unsentenced and awaiting trial), which is also punitive and highly disruptive. For example, 50% of women who entered prison in the UK in 2009 were on remand, often for minor offenses, and many did not receive a custodial sentence after trial (Epstein, 2011). In New South Wales (NSW) Australia, the context of this research, more than half (54%) of women in prison are on remand (BOCSAR, 2024).

Research shows that intersecting structural inequities and systemic health and social inequalities are the context of women’s incarceration (Gueta, 2020; Blankenship *et al.*, 2023; McCausland and Baldry, 2023). This is demonstrated by the over-representation of marginalised groups in prison. For example, racialised women are disproportionally represented in prison. Aboriginal and Torres Strait Islander [1] women make up approximately half (46%) of the female prison population in Australia, despite accounting for only 3% of the total female population (ABS, 2024). Similarly, First Nations women in Canada (McGuire and Murdoch, 2021) and Black, Latina and American Indian women in the USA (Heimer *et al.*, 2022; Wildeman and Wang, 2017) are vastly overrepresented in federal prisons. Lesbian or bisexual women (Simpson *et al.*, 2019), and women with a cognitive disability (Baldry, 2010; Dowse *et al.*, 2009), are over-represented in prison in Australia and globally (Jones, 2021; Donohue *et al.*, 2021). Incarcerated women are more likely to come from backgrounds of socio-economic inequality and exclusion, experiencing higher rates of poverty, unemployment and homelessness before their incarceration compared to men in prison (Bucerius and Sandberg, 2022; Heimer *et al.*, 2023; AIHW, 2020). The vast majority of women in prison are victims/survivors of gender-based violence (Australia’s National Research Organisation for Women’s Safety, 2020; Karlsson and Zielinski, 2020; St. Cyr *et al.*, 2021) and are at increased risk of violence-related death on release (Willoughby *et al.*, 2021). Approximately 80% of women in prison have a mental health diagnosis (World Health Organisation, 2021), which is frequently linked to lifetime experiences of trauma and abuse (Karlsson and Zielinski, 2020). Mental health and substance use are cumulative and compounding issues, as they often exacerbate the challenges faced by women both before, during and after incarceration (Anderson *et al.*, 2020; Bartels *et al.*, 2020).

“Gender-responsive” programs have emerged to address the specific needs of women in prison (Bloom *et al.*, 2003). Gender-responsive approaches assume that better outcomes will result by adapting existing systems and programs (which are male-centric) to suit women’s specific needs (Bloom *et al.*, 2003; Carlton and Segrave, 2016). A systematic review (Edwards *et al.*, 2022) looking at the efficacy of programs for women with substance use disorders internationally found that gender-responsive transitional programs (i.e. programs that provided pre- to post-release support) were observed to have greater reductions in recidivism for participants. Similarly, a meta-analytic review comparing the effectiveness of gender-responsive and “gender-neutral” programs at reducing recidivism for women found that when analysis was limited to higher quality studies, gender-responsive programs led to greater reductions in recidivism (Gobeil *et al.*, 2016). However, both reviews found that the included studies were lacking clear definitions for gender-responsive interventions, limiting the understanding of which elements were most effective (Edwards *et al.*, 2022; Gobeil *et al.*, 2016). Compounding this is that research on incarcerated women often involves small sample sizes because of the relatively lower number of women in prison compared to men, thereby limiting the generalizability of findings and the ability to draw robust conclusions about the needs of incarcerated women (Hidayati *et al.*, 2023; Van Hout *et al.*, 2022). To address these research gaps and to inform gender-responsive policy and practice, this study aims to investigate the services and supports women need when exiting prison using a large Australian sample over 10 years.

## Methods

This is a retrospective cohort study of women and men in prison who engaged with the prison health program “Connections” in NSW, Australia between January 1st 2008 and June 30th 2018. Of all states and territories in Australia, NSW has the largest prison population (ABS, 2024). Connections is a voluntary state-wide program, providing pre-release assessment and post-release linkage to health and welfare-related services for adults who are sentenced and self-identify as having problems with illicit drugs and/or alcohol. As this is a health program, clinical support workers (CSWs) refer to participants as patients. Full details of Connections can be found in the published program evaluation study protocol (Sullivan *et al.*, 2019).

Given the rates of reimprisonment, some patients have had more than one contact with Connections. As such, we conducted the analysis on patients’ first engagement with the program, regardless of their history of previous incarceration. Patients’ subsequent episodes and those who did not complete the pre-release assessment were excluded from the analysis.

### Data source

The data was sourced from the Connections database, which is an administrative dataset maintained by the Justice Health and Forensic Mental Health Network (JHFMHN), a branch of the state health service. Eligibility criteria for Connections are listed in Text Box 1. Eligibility between 2007 and 2015 included only those who identified as having an illicit drug use issue. Since 2015, Connections extended their eligibility to also include patients who self-reported problematic alcohol use. There was no difference in the prevalence of self-reported drug problem between years before this change and after, indicating perhaps many who had drug problems also had issues with alcohol.

**Text Box 1**: Eligibility for the Connections program 2007–2015

To be eligible for Connections, patients must be in an Adult Correctional Centre located in the state of NSW and:

1. self-report problematic substance use;
2. be sentenced on all charges at the time of referral to Connections (i.e. not on remand);
3. are due for release from prison within four weeks; and
4. meet one of the following clinical criteria:
  - currently on Opioid Substitution Therapy (OST);
  - ceased OST in the six months before release;
  - is pregnant or gave birth during the current period of incarceration or in the six months prior to entering custody and had a history of problematic substance use; or
  - not on OST but has engaged with treatment services specifically targeted at substance use disorders.

Baseline data were collected by CSW using the Initial Questionnaire (INQ) during face-to-face interviews. The INQ is administered generally no later than four weeks before the patient’s release from prison and is designed to assist CSWs to identify issues that may be relevant to the patient’s transition from prison to the community. Based on patient responses, CSWs recommend supports that might be beneficial and make links/referrals to appropriate community-based services before release from prison.

The INQ included demographic questions about life course and current: physical and mental health, suicide (considered or attempted), self-harm (other than attempted suicide, i.e. cutting, self-mutilation), substance use and social determinants of health (SDOH) such as education and literacy, employment history, housing arrangements and social support in the community. In addition, the INQ included three validated survey instruments: the Generalised Health Questionnaire-12 (GHQ-12); the Brief Treatment Outcome Measure-Concise (BTOM-C); and the mental health component of the Short Form-12 Health Survey (SF-12). The mental health component of the SF-12 has been demonstrated to be a reliable and valid use of self-reported mental health status (Larson, 2002; Ware *et al.*, 1996). The GHQ-12 is also used as a measure of current mental health. It is a well-known standardised health test for measuring minor psychological distress (Montazeri *et al.*, 2003). The BTOM-C is a multidimensional instrument administered at the commencement of treatment (or intervention) and at follow-up, to monitor treatment outcomes for participants receiving opioid pharmacotherapy and for use in treatment evaluation research (AIHW, 2006).

### Re-entry support variables

Using a combination of questions and validated tools from the INQ, six re-entry support variables were computed: substance use, housing, employment, mental health, social support and parenting. These variables represent areas of support and service provision that might be needed on release and were coded as *re-entry support need identified* (coded as 1) or *no re-entry support need identified* (coded as 0).

*Substance use support* was identified if patients reported having a drug problem before imprisonment; current offence was substance-related; stated they had problems with drugs during this incarceration or they might have difficulty remaining drug/alcohol free after release; or patients indicated that the people they spent most their time with or lived with before this incarceration also used illicit drugs on the BTOM-C.

*Housing support* was identified when patients stated they did not have stable housing or described a non-stable form of housing upon this release (i.e. psychiatric home, alcohol/drug treatment residence, shelter/refuge, caravan on serviced site and no usual residence/homeless) or they asked for help finding housing for release.

*Employment support* was identified when patients stated that before this imprisonment they have never been formally employed or they did not have a job to go to on release.

*Mental health support* was identified when patients shared that they have received treatment by a psychiatrist/doctor or Community Mental Health team for a mental health problem; been previously diagnosed with a mental health condition (such as depression, schizophrenia, bi-polar disorder, anxiety, personality disorder, alcohol dependence, drug dependence, attention-deficit/hyperactivity disorder [ADD/ADHD] and other mental illness); or scored ≥ 50 on the SF12 for mental health (indicating patients may need mental health support).

*Social support* was identified when patients did not list an emergency contact on the INQ or they stated they had difficulties keeping in contact with family/friends during their imprisonment (or suspected they would after release) or identified partner or relative conflict on the BTOM-C.

*Parenting support* was identified when patients stated they had children and they usually lived with those children or stated they would like to live with them after release. Patients who were parents were also identified for support if they said they currently did not have access to their children and, in the month before imprisonment, they had contact with child protective services, had a child restored or removed from their care on the BTOM-C.

### Statistical analysis

All data analyses was performed using IBM Statistical Package for the Social Sciences (SPSS) (version 27) software (IBM Corp Released, 2020). Frequencies with percentages for categorical variables and means with standard deviations or medians with interquartile ranges for continuous variables were calculated. Statistical differences between women and men were determined with Chi-square tests for categorical variables and independent-sample *t*-tests for continuous variables as appropriate. Statistical significance was set at the *p*-value < 0.05.

Multiple logistic regression model was used to assess the associations between sex (women) and re-entry support needs, adjusted for admission year, age, Australian born and Aboriginal and Torres Strait Islander status. We adjusted for admission year to account for program changes and improvements between 2008 and 2018. Other patient sub-groups (age, Australian born and Aboriginal and Torres Strait Islander status) were chosen, as the literature has shown that particular sub-groups may have different re-entry support needs depending on age (Maschi *et al.*, 2013), being from a culturally and linguistically diverse background (Ogloff *et al.*, 2022) and identifying as a Aboriginal and Torres Strait Islander person (Abbott *et al.*, 2018; Rose *et al.*, 2019). In response to the compounding effects of multiple incarcerations (Brinkley-Rubinstein, 2013), we also adjusted our analysis for first time in custody. Crude and adjusted odds ratios (AORs) and 95% confidence intervals (CIs) were estimated.

### Ethics

This project is embedded in a wider project titled *Recidivism, health and social functioning following release to the community of NSW prisoners with problematic drug use: an Evaluation of the Connections Program* (i.e. Connections). This is a National Health and Medical Research Council (NHMRC) funded project (GNT1109009). Ethics approval for this project was granted from NSW Population and Health Services Human Research Ethics Committee (HREC) (HREC/16/CIPHS/17); Aboriginal Health Medical Research Council HREC (1187–16); Justice Health and Forensic Mental Health Network HREC (Reference number: G217-16); Corrective Services NSW Ethics Committee (D190221581); University of Technology Sydney (ETH18-2587); and the University of Newcastle (H-2020–0074).

## Results

Records of 5,598 patients (54.7% of all study participants), who had their first engagement with Connections and completed the INQ, were included in this study. Of those, 18% (*n* = 989) were women and 82% (*n* = 4609) were men. Socio-demographic characteristics and incarceration history of the cohort by sex are shown in Table 1. Women were significantly younger than men (mean 33.5 years (SD = 7.8) vs 35.0 (SD = 8.4); *p* < 0.001) and a higher proportion of women identified as Aboriginal or Torres Strait Islander (41.1% vs 28.9%; *p* < 0.001), were partnered (29.3% vs 23.8%; *p* < 0.001), had children (76.8% vs 66.1%; *p* < 0.001), reported never having participated in any formal employment (30.2% vs 13.3%; *p* < 0.001), had previously considered/attempted suicide (35% vs 29%; *p* < 0.001) and have deliberately self-harmed (16% vs 13%; *p* = 0.014) compared to men. Similar rates of patients reported not completing high school (86.8% vs 87.5%; *p* = 0.05) and having a drug problem before this incarceration (85.9% vs 85.4%). Less women reported difficulties in reading, writing or counting money compared to men (12% vs 18%; *p* < 0.001).

**Table 1:** Socio-demographic characteristics and incarceration history by sex

Socio-demographic characteristics and incarceration history	Total n (%)^a^ n = 5,598	Sex *n* (%)a		*p*-value
		Women n = 989 (18%)	Men n = 4,609 (82%)	
Age; mean (SD)		33.5 (7.8)	35.0 (8.3)	<0.001
*Country of birth*				
Australia	4,878 (90.4)	865 (92.0)	4,013 (90.0)	0.06
Overseas	521 (9.6)	75 (8.0)	446 (10.0)	
*Aboriginal and Torres strait islander status*				
Indigenous	1,637 (31.0)	371 (41.1)	1,266 (28.9)	<0.001
Non-Indigenous	3,640 (69.0)	531 (58.9)	3,109 (71.1)	
*Highest level of education*				
No school certificate (did not complete high-school)	4,751 (87.4)	837 (86.8)	3,914 (87.5)	0.05
High school certificate (completed high-school)	315 (5.8)	70 (7.3)	245 (5.5)	
Tertiary education (post high school qualifications)	371 (6.8)	57 (5.9)	314 (7.0)	
*Difficulties reading, writing or counting money*				
Yes	880 (16)	110 (12)	770 (18)	<0.001
No	4,490 (84)	830 (88)	3,660 (83)	
*Employment history*				
Have been previously employed	4,487 (83.7)	651 (69.8)	3,836 (86.7)	<0.001
Never previously employed	872 (16.3)	281 (30.2)	591 (13.3)	
*Marital status*				
Never married	3,368 (62.7)	570 (61.0)	2,798 (63.1)	<0.001
Married/defacto/regular partner	1,327 (24.7)	273 (29.2)	1,054 (23.8)	
Divorced/separated/widowed	676 (12.6)	91 (9.7)	585 (13.2)	
*Children*				
Yes	3,637 (68.0)	717 (76.8)	2,920 (66.1)	<0.001
No	1,715 (32.0)	217 (23.2)	1,498 (33.9)	
*Suicide (considered or attempted)*				
Yes	1,603 (30)	325 (35)	1,278 (29)	<0.001
No	3,765 (70)	607 (65)	3,158 (71)	
*Deliberately self-harmed (other than attempted suicide) including slashing, self-mutilation, etc.*				
Yes	747 (14)	154 (16)	593 (13)	0.014
No	4,640 (86)	786 (84)	3,854 (88)	
*Drug problem (before current incarceration)*				
Yes	4,613 (85.5)	808 (85.9)	3,805 (85.4)	0.72
No	783 (14.5)	133 (14.1)	650 (14.6)	
*History of incarceration*				
Yes, previously incarceration	4,807 (89.3)	749 (79.6)	4,058 (91.4)	<0.001
No, first time in prison	574 (10.7)	192 (20.4)	382 (8.6)	
*Current offence was alcohol or drug related*				
Yes	4,038 (75.1)	699 (74.4)	3,339 (75.3)	0.60
No	1,338 (24.9)	240 (25.6)	1,098 (24.7)	
*Length of current sentence*				
Less than 6 months	1,423 (26.5)	409 (44.3)	1,014 (22.9)	<0.001
6–12 months	1,926 (35.9)	328 (35.5)	1,598 (36.0)	
1–4 years	1,533 (28.6)	172 (18.6)	1,361 (30.7)	
4–10 years	404 (7.5)	9 (1.0)	395 (8.9)	
10 years or more	74 (1.4)	5 (0.5)	69 (1.6)	

Note(s):

^a^Those who did not answer (i.e. not stated or refused to answer) were removed from analysis; as a result, total numbers do not equal total number of patients but total number of people who answered each question

**Source(s):** Table by author

More women were in prison for the first time (20.4% vs 8.6%; *p* < 0.001) compared to men. High proportions of patients were in prison for substance-related offences (74.4% vs 75.3%; *p* = 0.60). There were significant differences between women and men and current prison sentence length. Nearly double the proportion of women were sentenced to less than six months compared to men (44.3% and 22.9%; *p* < 0.001). Almost twice the proportion of men were sentenced to one year or more compared to women (41.2% and 20.1%; *p* < 0.001).

### Re-entry support

The re-entry support needs identified for women and men are reported in Table 2. Using the variables as a measure of “re-entry support need”, women needed more re-entry support for housing (52.3% vs 46.1%; *p* < 0.001), employment (87.6% vs 78.6%; *p* < 0.001) and parenting (68.3% vs 60.1%; *p* < 0.001) compared to men. Rates of “re-entry support need” were similar for women and men on the substance use (88.3% vs 89.5%; *p* = 0.26) and mental health (75.7% vs 76.0%; *p* = 0.26) variables.

**Table 2:** Re-entry support needs identified by sex

Re-entry support needs	Total *n* (%) n = 5,598	Sex *n* (%)		*p*-value
		Women n = 989 (18)	Men *n* = 4,609 (82)	
Substance use	4,997 (89.3)	873 (88.3)	4,124 (89.5)	0.26
Mental health	4,249 (75.6)	749 (75.7)	3,500 (76.0)	0.88
Housing	2,641 (47.2)	517 (52.3)	2,124 (46.1)	<0.001
Employment	2,268 (80.2)	866 (87.6)	3,620 (78.6)	<0.001
Social	1,891 (33.8)	352 (35.6)	1,539 (33.4)	0.19
Parenting	3,443 (61.5)	675 (68.3)	2,768 (60.1)	<0.001

**Source(s):** Table by author

### Multiple logistic analysis

The multiple logistic regression, adjusting for sociodemographic factors and first time in custody, found that being a woman was associated with increased odds of “re-entry support need” in housing (1.51 AOR; 95% CI: 1.29–1.77; *p* < 0.001), employment (2.97 AOR; 95% CI: 2.26–3.91; *p* < 0.001) and parenting (1.68 AOR; 95% CI: 1.41–2.00; *p* < 0.001) when compared to men (Table 3).

**Table 3:** Odds ratio of correlates of women compared to men and re-entry support areas

Re-entry support needs	Adjusted odds ratio^a^	(95% CI)	*p*-value
Substance use	0.98	(0.73–1.32)	0.91
Mental health	1.15	(0.95–1.39)	0.16
Housing	1.51	(1.29–1.77)	<0.001
Employment	2.97	(2.26–3.91)	<0.001
Social	1.07	(0.91–1.26)	0.42
Parenting	1.68	(1.41–2.00)	<0.001

Note(s):

^a^Adjusted for admission year, age, Australian born, Indigenous status and first time in custody

**Source(s):** Table by author

## Discussion

Key areas of re-entry support for women were housing, employment and parenting. These results were reproduced by the multivariate regression, where being a woman was associated with increased odds of “re-entry support need” in housing by 51%, threefold increased odds for employment and 68% increase for parenting. These findings highlight critical and specific areas for the development of gender-responsive programs and frameworks; they also need to be understood in the context of broader intersecting health and social inequities experienced by women.

Reinforcing previous research, we argue that safe and stable (long-term) housing on release from prison is foundational for improving a women’s ability to survive in the community after release. Providing stable housing to people exiting prison has been shown to improve mental health outcomes, significantly reduce substance use and improve health utilisation after release (Kouyoumdjian *et al.*, 2015; Tweed *et al.*, 2021). Moreover, safe and stable housing provides an environment for reuniting with children and enables women to pursue other needs such as finding a job or participating in job training (Salem *et al.*, 2021; KWOOP, 2020b; Baldry and McCausland, 2009). These are SDOH and social determinants of incarceration for women who have experienced imprisonment, with previous research finding that absence of stable post-release housing, financial problems, domestic violence and statutory removal of children, are associated with worsening mental health, substance uptake after release from prison and recidivism (Edwards *et al.*, 2024).

Employment plays a critical role in women’s survival. Employment not only provides an income but also can provide a sense of meaningful or gratifying purpose (*KWOOP*, 2020a; *van den Broek et al.*, 2021). The threefold increased odds for employment support among women is consistent with the literature, as one of the most frequently reported re-entry needs for women is assistance in finding and keeping a job (O'Brien and Leem, 2007). Securing a job is extremely difficult for people exiting prison (Nally *et al.*, 2014), and employers tend to regard recently released women more negatively than men (Sheppard and Ricciardelli, 2020). In addition to the stigma of prison and/or having a criminal record, women are also subjected to gendered inequities that can prevent them from accessing employment services and labour market opportunities and, subsequently, are at greater risk of poverty and being forced to return to criminal activity (Butcher *et al.*, 2017). The findings of our study underscore the need for options in relation to job placement, skill-building programs and employment pathways as part of ‘gender-responsive’ programs and practice approaches.

While both imprisoned women and men can experience parental distress when separated from their children (Breuer *et al.*, 2021; Bartlett *et al.*, 2018), the parenting issues of women and men differ in that the majority of women are usually the primary and sole carer of children before incarceration. In most cases when a father is imprisoned, their children remain in the care of their mother. The children of imprisoned mothers are eight times more likely to be placed in foster care and seven times more likely to be placed in a group home or an institutional setting when compared to the children of imprisoned fathers (Kennedy *et al.*, 2020). The imprisonment of mothers, therefore, has significantly more harmful impacts on children’s well-being.

For mothers exiting prison whose children have been taken into “out of home care” by statutory child protection services, studies have shown there are numerous barriers to regaining custody. For example, there is not only a sheer paucity of affordable or government subsidised (i.e., public or social) housing that can accommodate a family (wait times for three-bedroom homes currently exceed 10 years) (Stone, 2013; Acosta and Gartland, 2022); mothers are typically not classified as high priority for public/social housing because children are not currently in their care (Stone, 2013). Moreover, mothers often need to show stable employment; however, caregiving responsibilities can often conflict with job requirements (e.g., working full time and lack of flexibility to accommodate school hours), making it difficult to maintain steady employment (Roddy, 2023). It can, thus, be seen that housing, employment and parenting “need” are interrelated, as women face structural and systemic barriers to all three, and the attainability of each is at least partly dependent on the attainability of the others. This paradoxical situation is a further punishment and an injustice, as women are essentially denied access to health and well-being in the community because of their history of incarceration. “Gender-responsive” policy should, therefore, involve the implementation of strategies that address these barriers in an intersecting way, for example, by establishing affordable housing and plans for reconnection with children before release. Without this support, mental health may worsen after release, potentially influencing substance use and positioning women at increased risk of harm and reoffending (Edwards *et al.*, 2024).

Although rates of mental health “re-entry support need” were similar for women and men in this study, rates were high, and it is important to consider how women’s social determinants of mental health and other health issues may be different to men’s post-release. Many women with mental health diagnoses, often rooted in trauma from gender-based violence, cite using substances to alleviate mental health symptoms (McCausland and Baldry, 2023; Johnson *et al.*, 2013; Langan and Pelissier, 2001; Stalans, 2009). However, this cycle can exacerbate symptoms, leading to worsening mental health and greater severity of substance dependence (Jamin *et al.*, 2021). Moreover, substance use is associated with SDOH such as homelessness and unemployment and further victimisation (i.e. gender-based violence) (Jamin *et al.*, 2021; Johnson *et al.*, 2013; Saxena *et al.*, 2016). This suggests that gender-responsive approaches need to both respond to and look beyond mental health scores on exiting prison to what women are saying about their social circumstances and what they know to be the risks and harms in relation to their substance use. This could include concerns about money/income, homelessness, unemployment, children and gender-based violence.

Consistent with previous research (Hawton *et al.*, 2014; Janca *et al.*, 2022), a higher proportion of women reported previous self-harm and previous suicidal thoughts or suicide attempts. This contradicts evidence from general population studies in which rates of suicide are typically higher among men than women (Naghavi, 2019), emphasising the vulnerability of women on exiting prison. One of the commonly cited medical and psychological factors underpinning self-harm among women is in response to trauma (Gurung, 2018; McLafferty *et al.*, 2019; McManus *et al.*, 2019; Walker *et al.*, 2021; Stewart *et al.*, 2018). In addition, women recently released from prison experience substantially higher rates of socioeconomic disadvantage, substance use and mental illness, which are all established risk factors associated with suicide and self-harm (King *et al.*, 2018; Fries *et al.*, 2014; Borschmann *et al.*, 2017; Gunnell and Chang, 2016; Lorant *et al.*, 2005). As above, the impact of these risk factors could be minimised by (gender-responsive) programs that provide integrated assistance with housing, accessing income support and mental health and substance use support. Interviews with women survivors of near fatal self-harm in prison have suggested that programs should include purposive activity which can offer a positive distraction from self-harm and suicidal thoughts (Borrill *et al.*, 2005).

A high proportion of women and men in our study attributed their current imprisonment to substance-related offences, which was unsurprising given our study population (participants on a program for adults exiting prison with a substance use problems). However, twice the number of women were imprisoned for the first time and double the proportion of women were sentenced to less than six months compared to men. Considering sentence length is usually influenced by offence seriousness and risk to the general public (Hedderman and Gunby, 2013), we can, therefore, assume that women were sentenced for minor drug offences in comparison to men and did not pose a threat to the community. Some judges may feel short sentences are a more lenient option for women, especially in the absence of viable alternatives; however, this perception is in contrast with the evidence around the disproportionate harms short sentences can have on women. Short sentences typically exclude women from access to rehabilitation programs in prison and, therefore, compound women’s disadvantages on release (Cracknell, 2018). Prison has direct devastating impacts in the form of loss of housing, loss of employment, children being removed and taken into the out of home care, stigmatisation and further marginalisation (Australian Law Reform Commission, 2017). Acknowledging the personal and intergenerational harms caused by incarceration, especially the incarceration of women, we argue that the criminal legal system needs to be gender-responsive and provide women with non-punitive, supportive options in the community. There are exemplars of diversionary programs that can be drawn upon to this end (Abrams *et al.*, 2020). This will reduce the number of women being imprisoned and prevent the harmful impacts of women’s imprisonment from occurring, including reducing recidivism.

### Strengths and limitations

A major strength of this study is drawn from its large sample size, which is rare. This study extended our knowledge of women’s health and social circumstances on exiting prison and the types of assistance that might be useful in a large Australian sample over 10 years. As Connections focused on patients with substance use issues, our findings may not be generalizable to the wider women’s prison population. However, considering most women in prison report histories of substance-use and poor mental health, the results of this study can potentially be applied to a larger number of women in prison in Australia and worldwide.

A number of study limitations should be considered when interpreting these results. Firstly, not all questions in the INQ asked directly “what do you need”; therefore, the results of this analysis are qualified in their interpretation of the services and supports that women need on release from prison. Nonetheless, our statistical results reinforce existing qualitative research on transitional support for women, which indicates stable housing, employment and parenting related support are the most critical re-entry needs for women exiting prison (Salem *et al.*, 2013; Kendall *et al.*, 2018; O'Brien and Leem, 2007; Bloom *et al.*, 2003; Breuer *et al.*, 2021).

Secondly, the INQ was designed as a comprehensive pre-release assessment to capture the health and well-being needs of adults in prison with histories of substance use to facilitate community referral. The majority of assessment tools used within the criminal legal system have been largely derived from non-Aboriginal male-based needs and systems (Salisbury *et al.*, 2009). We acknowledge that the unique needs of women may not have been captured by the data. Moreover, there is an absence of intersectional analysis as this was outside the scope of the study. While our results show the differences between re-entry support needs for women and men, we acknowledge that the unique needs of Aboriginal and Torres Strait Islander women, women from ethnic minority community groups and LGBTQI+ women have not been captured.

Finally, all data collected were self-reported and because of the sensitive nature of many questions (i.e. housing on release, histories of substance use and self-harm/suicide, etc.), the responses to these questions could be subject to reporting bias because of stigmatisation or fear. As a result, there is a risk of under-reporting on the INQ by women, who may not identify an issue in fear it might jeopardise their release, reunification of their children or because of the stigma around mental health and substance use (van Olphen *et al.*, 2009; Stone, 2015).

### Future research implications

Our findings indicate that at the point of release from prison women identify for support with housing, employment and parenting, while also struggling with intersecting challenges related to substance use, mental health and the impact of short custodial sentences. Building upon these findings, future research should centre the voices of women who have exited prison, asking them directly about their needs, what is helpful and the barriers to services and support they experienced during transition from prison to community. By delving further into the post-release experiences of women who have been incarcerated, we can enhance our understanding of what “gender-responsiveness” policy and practice should entail and improve systems for women in ways that prevent recidivism. Moreover, there should be specific qualitative research with Aboriginal and Torres Strait Islander women, women from ethnic minority communities and trans and gender diverse women to understand their experiences with services and what “gender responsiveness” looks like for them.

Our findings also indicate that research is required with judicial officers and lawyers regarding the impacts of short custodial sentences on women and decision-making around community-based options. Interpretations of the law and court practices can be changed, and enhancing understandings of the gendered impacts of the law could be a promising way to prevent the incarceration of women in the first place.

## Conclusion

This research informs understandings of women’s health and social circumstances on exiting prison and the types of assistance that might be useful. Specifically, this large-scale study of 10 years of routinely collected data found that twice the proportion of women were incarcerated for the first time and double were sentenced to less than six months compared to men. At the point of release, women needed greater support with housing, employment and parenting compared to men. Our findings indicate an approach to “gender-responsiveness” that is defined by addressing women’s social determinants of health and incarceration, including supportive diversion options in the community for women and mothers. This requires policy, service provision and practice that will support women and address broader gender inequity taking an intersectional approach.

## Note

[1.] In this article, we use the term “Aboriginal and Torres Strait Islander” to refer to First Nations peoples in Australia, in alignment with government reporting and public health guidelines in Australia.
